# On the Entropic Characterization of Mayonnaise Processing

**DOI:** 10.3390/e28080879

**Published:** 2026-08-05

**Authors:** Lijesh Koottaparambil, Roger A. Miller, Michael M. Khonsari

**Affiliations:** 1 Department of Mechanical Engineering and Industrial Engineering, Louisiana State University, Baton Rouge, LA 70803, USA; lijesh@lsu.edu; 2 M Consulting LLC, 18342 Char A Banc, Baton Rouge, LA 70817, USA; ramiller@mconsultingla.com

**Keywords:** mayonnaise, accumulated entropy generation, motor current monitoring, viscosity, shear-induced degradation

## Abstract

Mayonnaise is a high-viscosity food emulsion whose consistency evolves during shearing due to structural rearrangement and possible emulsion destabilization. This study presents a laboratory-scale proof-of-concept for adapting an established motor current-derived accumulated entropy generation (AEG) framework as a thermodynamic descriptor for monitoring mayonnaise structure changes. First, eight reference fluids were tested using a rotating-bob viscometer at shear rates of 600, 800, and 1000 s^−1^ to establish the relationship between viscosity and motor current. The corrected current response showed a strong linear correlation with viscosity. The approach was then extended to commercially available mayonnaise samples. Due to the higher viscosity and structured nature of mayonnaise, testing was performed at 1000 s^−1^, where stable shearing could be achieved. A modified impeller-based viscometer setup was used to continuously shear the mayonnaise and monitor the motor current in situ, while rheometer measurements were performed independently to validate the corresponding viscosity changes during shearing. The motor current decreased with shearing time, consistent with the reduction in measured viscosity. The calculated AEG increased continuously and distinguished the shear stability of different mayonnaise formulations. The viscosity degradation rates of two different mayonnaises are characterized using the degradation coefficient *B* introduced in the degradation–entropy generation (DEG) theorem. A higher *B* value indicates greater structural breakdown. These results suggest that current-derived entropic parameters (*B* coefficient and AEG) may serve as practical, sensor-accessible descriptors for monitoring mayonnaise consistency evolution when direct torque measurement or in-line rheology is unavailable.

## 1. Introduction

Mayonnaise is a concentrated oil-in-water emulsion whose final quality is governed by the formation, stability, and deformation behavior of a densely packed droplet network. Conventional mayonnaise is typically composed of a high oil fraction dispersed in an aqueous phase containing egg-based emulsifiers, acidulants, salt, and other stabilizing ingredients. The egg yolk components adsorb at the oil–water interface and help stabilize the dispersed oil droplets, while the continuous phase and any added hydrocolloids influence viscosity, yield stress, spreadability, and storage stability [1,2,3]. Because of this microstructural complexity, mayonnaise does not behave as a simple Newtonian liquid; it commonly exhibits shear-thinning behavior, yield-stress response, viscoelasticity, thixotropy, and time-dependent structural evolution under shear [4,5,6]. These rheological characteristics are closely associated with product perception, stability, spreadability, and resistance to phase separation.

During mayonnaise manufacturing, mechanical shearing plays a central role in dispersing the oil phase, reducing droplet size, developing the emulsion structure, and producing the desired consistency. Processing variables such as mixing speed, impeller geometry, homogenization intensity, oil addition history, temperature, ingredient composition, and emulsifier functionality can strongly affect droplet-size distribution, viscosity, yield stress, and emulsion stability [7,8,9]. Insufficient shearing may lead to poor droplet dispersion and weak structure development, while excessive or unsuitable shearing can promote structural breakdown, viscosity loss, or oil separation. Therefore, mayonnaise production requires not only the correct formulation but also control over the mechanical pathway by which the emulsion structure is formed and maintained.

Rheological characterization is commonly used to evaluate mayonnaise quality because viscosity, yield stress, and viscoelastic properties provide information about the resistance of the emulsion to deformation [3,10,11,12]. However, conventional rheometer measurements are generally performed off-line or at-line using collected samples. Although such measurements are accurate and useful for quality validation, they do not provide continuous information about how the product evolves during actual processing. This limitation is important because mayonnaise structure can change rapidly during shearing, and the final rheological state depends on the complete processing history rather than only the final formulation. In industrial systems, direct in-line rheometry or torque measurement may also be difficult to implement due to hygienic design, equipment geometry, cost, and disruption to existing production lines.

A more practical approach to process monitoring is to use sensor signals already accessible from rotating equipment. In mixers, homogenizers, and impeller-driven systems, the mechanical resistance of the product changes the motor load. This change can be reflected in torque, power, or motor current. Recent work in food processing has shown that motor current signals can be used to monitor dough development and identify changes in material resistance during kneading [13], while broader process analytical technology studies emphasize the value of real-time sensor signals for replacing delayed end-point testing with in-process control [14,15]. Similar sensor-integrated modeling concepts have also been adopted in other engineering systems, including digital-twin frameworks for full-field structural assessment and prediction, and energy-based optimization of electrified turbocharging systems where transient response and energy consumption must be jointly evaluated [16,17]. For mayonnaise, this idea is attractive because the product’s viscosity and structure influence the load experienced by the rotating element. Thus, motor current may provide a simple, non-invasive, and low-cost indicator of evolving shear resistance during processing.

However, current or torque alone remains an instantaneous process signal. It indicates the load at a given time, but it does not by itself quantify the cumulative mechanical history experienced by the material. This is a critical limitation for structured emulsions such as mayonnaise, where deformation, droplet rearrangement, viscous dissipation, and possible destabilization accumulate over time. A physics-based descriptor is therefore needed to convert time-dependent process signals into a measure of irreversible material evolution. Irreversible thermodynamics provides a reliable framework because mechanical shearing dissipates energy through viscous flow, internal friction, droplet deformation, and structural rearrangement. The entropy generated during shearing can accumulate over time to yield accumulated entropy generation (AEG), which represents the cumulative irreversible dissipation experienced by the material. Previous studies have shown that entropy generation can serve as a degradation or damage descriptor in irreversible material systems, including fatigue, tribological degradation, composites, and metals under unidirectional stress, Li-ion batteries, metals degrading due to hydrogen embrittlement and corrosion, and mechanically sheared grease [7,18,19,20,21,22,23,24,25,26].

The degradation–entropy generation (DEG) [27] theorem provides a quantitative framework for relating material degradation to entropy generation. According to this theorem, a degradation measure, w, is proportional to the accumulated entropy generation, with the proportionality depicted by the degradation coefficient, B. Therefore, B represents the degradation produced per unit accumulated entropy generation. This coefficient has been applied across different degradation problems, ranging from electrical and mechanical systems to chemically driven degradation processes, to compare the rate at which materials deteriorate under irreversible loading conditions [28,29,30,31]. A higher B value indicates greater degradation for a given amount of generated entropy, whereas a lower B value reflects greater resistance to degradation.

Of particular relevance is the recent extension of AEG to lubricating grease manufacturing, where real-time motor current, voltage, temperature, and batch-volume data were used to calculate AEG during industrial grease production. In that work, AEG was shown to correlate with final grease consistency and to provide an in-situ process-monitoring variable for batch control [32,33]. Like mayonnaise, grease [34] is a structured, semi-solid, multiphase material whose consistency evolves under mechanical shearing. The key difference is that, in mayonnaise, the thermodynamic response is associated not only with lubricant degradation but also with emulsion resistance, structural evolution, viscosity loss, and possible destabilization during shearing.

The present study evaluates the applicability of current-derived AEG and the effective DEG-based B coefficient for mayonnaise as a representative high-viscosity food emulsion. Since an industrial mayonnaise production unit was unavailable, a systematic laboratory-scale procedure was followed to establish the sensing principle in stages. First, the rotating-bob viscometer was used with eight selected reference fluids to measure viscosity at shear rates of 600, 800, and 1000 s^−1^. These initial fluid tests provided a controlled viscosity range and were used to determine whether the motor current response could be correlated with fluid viscosity under known shearing conditions. After establishing the current–viscosity relationship using the reference fluids, the same current-based monitoring approach was extended to four commercially available mayonnaise samples. Because of the higher viscosity and structured nature of mayonnaise, smooth and stable rotation between the bob and cup could not be consistently achieved at lower shear rates. Therefore, the mayonnaise measurements were performed at 1000 s^−1^, where stable shearing was obtained. The initial viscosity response of each mayonnaise sample at this shear rate was used as the reference value for comparison with the corresponding motor current response. Finally, to evaluate the time-dependent AEG response during mayonnaise shearing, the standard rotating-bob geometry was replaced with a 3D-printed impeller. This modification was introduced to provide a controlled laboratory-scale shearing configuration for evaluating the motor current and AEG responses of mayonnaise. The custom mixing impeller was not intended to reproduce the complete flow field of an industrial mixing system. In this impeller-based setup, the motor current was continuously recorded during shearing, while mayonnaise samples were collected at selected time intervals and independently tested using a rheometer. This stepwise approach—reference fluids, mayonnaise viscosity comparison, and impeller-based continuous shearing—provided a basis for evaluating whether current-derived AEG can be used as a thermodynamic descriptor of mayonnaise structure evolution.

The objective of this work is to evaluate the feasibility of using current-derived AEG as a thermodynamic descriptor for monitoring shear-induced structural changes in mayonnaise. Specifically, the study evaluates whether motor current can be correlated with viscosity, whether the current signal can track time-dependent viscosity reduction during shearing, and whether the resulting AEG response can distinguish mayonnaise formulations with different shear stability. In addition, the study determines the DEG-based B coefficient for two mayonnaise formulations to quantify their degradation rates in the initial and steady-state shearing regions. By connecting motor current response, rheometer-measured viscosity, and entropic parameter (*B* coefficient and AEG), this work provides an initial proof of concept for using current sensors to monitor shear-induced consistency changes in mayonnaise when direct torque measurement or in-line rheological monitoring is not available.

## 2. Materials and Methods

### 2.1. Theory for Current-Derived AEG During Mayonnaise Shearing

Mechanical shearing of mayonnaise involves viscous dissipation, droplet deformation, interfacial rearrangement, and changes in the emulsion structure. The thermodynamic relations used here are based on the previously established accumulated entropy generation and degradation–entropy generation frameworks [32,35,36], which are adapted in the present study to continuous mayonnaise shearing using motor current as a sensor-accessible indicator of evolving mechanical resistance. The mechanical power supplied during shearing may be distributed among irreversible viscous dissipation, temporary elastic or interfacial energy storage, structural rearrangement, and losses within the experimental system. Under the present continuous shearing conditions, kinetic energy accumulation was assumed to be negligible. Accordingly, Equation (1) represents only the contribution of irreversible viscous dissipation to entropy generation. For simple shear at approximately uniform temperature, the corresponding volumetric entropy-generation rate can be expressed as:

(1) $$\begin{array}{cccc} & {\dot{s}}_{g}=\frac{\tau \cdot \dot{\gamma }}{T}, & & \end{array}$$

where ${\dot{s}}_{g}$ is the volumetric entropy-generation rate, τ is the shear stress, $\dot{\gamma }$ is the shear rate, and T is the absolute temperature. The product $\tau \cdot \dot{\gamma }$ represents the irreversible viscous-dissipation rate per unit volume. It does not include recoverable mechanical-energy storage or losses outside the sample. Accordingly, when $P_{diss}$ denotes only the mechanical power irreversibly dissipated within the sample, Equation (1) may be written as,

(2) $$\begin{array}{cccc} & {\dot{s}}_{g}=\frac{P_{diss}}{T\cdot V_{s}}. & & \end{array}$$

The volume-normalized accumulated entropy generation over a shearing interval from 0 to t is therefore

(3) $$\begin{array}{cccc} & AEG\left(t\right)=\int_{0}^{t}\frac{P_{diss}\left(t\right)}{T\left(t\right)\cdot V_{s}}\;dt. & & \end{array}$$

In the present experimental arrangement, torque and shaft power were not measured directly. Instead, the motor current response was used as a sensor-accessible indicator of the relative change in energy input associated with the resistance of the mayonnaise during shearing. Under identical equipment, impeller geometry, operating speed, sample volume, and operating conditions, the measured current is proportional to the mechanical resistance encountered by the impeller. Consequently, the current-derived accumulated response can be written directly in terms of the measured current, applied voltage, temperature, and sample volume, without explicitly introducing motor power factor or motor efficiency. Since the same experimental configuration was maintained for all tests, these constant system characteristics are inherently embedded in the comparative response and do not influence comparisons between formulations.

Accordingly, the calculated quantity is interpreted as a current-derived comparative accumulated entropy-generation (AEG) response rather than an absolute thermodynamic entropy measurement. This approach enables direct comparison of structural evolution between mayonnaise formulations under identical testing conditions while avoiding the need for independent calibration of motor-specific electrical parameters.

For the time-dependent mayonnaise tests, the current decreased from its initial value as the viscosity and mechanical resistance of the sample decreased. To quantify this evolving response relative to the initial shearing condition, the current change was defined as:

(4) $$\begin{array}{cccc} & \Delta I\left(t\right)=I_{0}-I\left(t\right), & & \end{array}$$

where $I_{0}$ is the current measured at the beginning of shearing and $I\left(t\right)$ is the time-dependent current. The corresponding accumulated change-based current-derived response was calculated as

(5) $$\begin{array}{cccc} & {AEG}_{\Delta I}\left(t\right)=\int_{0}^{t}\frac{V_{m}\cdot \Delta I\left(t\right)}{T\left(t\right){\cdot V}_{s}}\;dt. & & \end{array}$$

Equation (5) represents the accumulated change in the current-derived dissipation response relative to the initial condition. Here, $V_{m}$ is the applied motor voltage, $\Delta I\left(t\right)$ is the time-dependent change in measured current, *T*(*t*) is the room temperature (25 °C), and $V_{s}$ is the mayonnaise volume of $1.7\times 10^{-5}\;m^{3}$ for each test. It is therefore used as a comparative descriptor of the cumulative evolution of mayonnaise resistance during shearing. The resulting AEG trajectory was then compared with rheometer-measured viscosity to evaluate whether motor current can capture the structural evolution of mayonnaise during processing. The present analysis assumes approximately constant line voltage (110 V), temperature, and sample volume under the applied operating conditions. For future applications, the sensing arrangement should be selected according to motor type, since AC and DC motors require different current measurement approaches.

In the present study, the mayonnaise was subjected to continuous shearing without an intermediate relaxation period, consistent with a continuously operating processing condition. Because the parameter is evaluated under dynamic conditions, it is not intended to represent a recovered or purely irreversible thermodynamic damage state. Therefore, the DEG concept is applied by relating the viscosity change, Δη, to AEG. Accordingly, the normalized viscosity change, $w=\Delta \eta /\eta_{0}$, was adopted as a process-relevant degradation indicator representing the apparent reduction in structural resistance during continuous shearing. It was defined as

(6) $$w\left(t\right)=\frac{\eta_{0}-\eta \left(t\right)}{\eta_{0}}=\frac{\Delta \eta \left(t\right)}{\eta_{0}},$$

where $\eta_{0}$ is the initial apparent viscosity and $\eta \left(t\right)$ is the apparent viscosity after a specified shearing duration. Within the DEG framework, the incremental change in the degradation parameter can be related to the entropy generation per unit volume as

(7) $$dw=B\cdot dS_{g}$$

where B is the viscosity-based degradation coefficient and $S_{g}$ is the entropy generation per unit volume. Assuming that the mechanism of degradation is approximately constant over the investigated shearing interval, the damage and entropy generation are related by:

(8) $$w(t)=B\cdot S_{g}(t)=B\cdot AEG(t).$$

Substituting $w_{f}=\Delta \eta /\eta_{0}$ and the current-derived AEG expression Equation (5) in Equation (8) yields.

(9) $$\begin{array}{cccc} & \frac{\Delta \eta }{\eta_{0}}=B\int_{0}^{t_{f}}\frac{V_{m}\cdot \Delta I\left(t\right)}{T\left(t\right)\cdot V_{s}}\;dt, & & \end{array}$$

Equation (9) represents the current-derived accumulated entropy-generation response used in the present study. The formulation assumes that the same motor, electrical configuration, impeller geometry, operating speed, immersion depth, and sample-loading procedure were maintained across the comparative tests. The normalized apparent-viscosity change was adopted as the damage variable, w, and the effective degradation coefficient was determined from the slope of the w—AEG relationship. Accordingly, *B* represents the increment in viscosity-based damage per unit current-derived AEG under continuous shearing. At the initial reference condition, both w and the change-based AEG are zero, and the relationship therefore originates at w = AEG = 0. If no viscosity-based damage develops, dw = 0, the corresponding degradation coefficient is zero. Because the current-to-mechanical-power conversion was not independently calibrated, the calculated *B* values are interpreted as comparative parameters specific to the present experimental configuration and are used to compare the shear stability of the two mayonnaise formulations.

### 2.2. Experimental Setup

#### 2.2.1. Rotating-Bob Viscometer Setup for Liquid/Oil Viscosity Measurement

**Figure 1:**
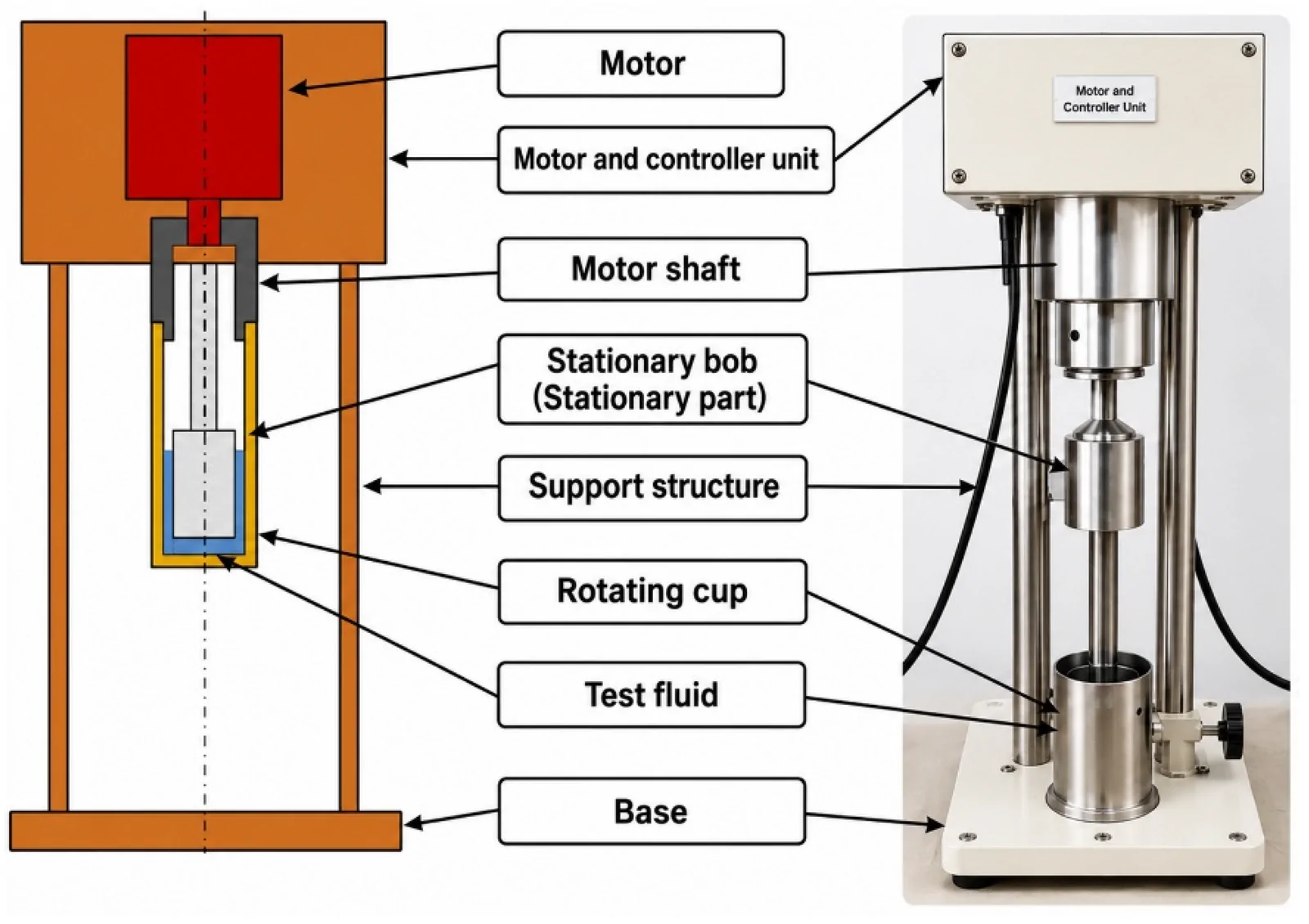
Rotating-bob viscometer used for viscosity measurement of reference fluids. The figure shows a schematic representation and a photograph of the experimental setup, including the motor and controller unit, motor shaft, stationary part, support structure, rotating cup, test fluid, and base.

The viscosity of the eight representative fluids was measured using a conventional rotating-bob viscometer, as shown in Figure 1. The instrument consists of a motor-driven spindle connected to a cylindrical bob that rotates within a stationary sample cup. During measurement, the fluid sample was in the rotating cup, and the cup was fastened to the motor shaft. The relative motion between the rotating cup and bob shears the fluid in the gap between them, allowing the sample’s viscosity to be determined.

These initial measurements were performed on low-to-medium viscosity fluids, including water, mineral oil, coolant, vegetable oil, lubricating oils, and selected oil blends. Three fixed rotational shear rates of 600, 800, and 1000 s^−1^ were used as the standard operating condition for the initial viscosity measurements. This speed was selected because the tested fluids were primarily Newtonian or near-Newtonian liquids, for which viscosity is expected to remain nearly independent of shear rate under laminar conditions. The experiments were performed on 50 mL of fluid. The bob was allowed to rotate until a stable reading was obtained, and the viscosity was recorded in centipoise (cP).

The AC motor current was measured using a CR4120S true-RMS split-core current transducer with a standard 4–20 mA output. The split-core configuration enabled non-invasive installation around the motor conductor, while the true-RMS measurement supported reliable monitoring of the AC-current response. The viscometer was also equipped with an integrated thermocouple, and temperature was continuously monitored during testing. The measured temperature remained close to room temperature with negligible variation; therefore, a separate temperature profile is not presented.

The details of the fluids considered for the testing are provided in Table 1. The fluids were classified using representative physical and chemical descriptions.

#### 2.2.2. Rheological Measurement of Commercially Available Mayonnaise Samples

Following the initial fluid measurements, the study was extended to commercially available egg-based mayonnaise samples. Four mayonnaise products were selected to evaluate the applicability of the current-based monitoring approach to high-viscosity food emulsions. Based on the product formulation, three of the selected samples were palm-oil-based mayonnaise products, while Mayo 4 was olive-oil-based. Therefore, the selected samples allowed comparison between mayonnaise formulations with similar product type but different oil-phase composition.

Compared with the initial liquid/oil samples, the mayonnaise samples exhibited substantially higher viscosity and stronger resistance to deformation, particularly at the beginning of shearing. At operating conditions below 1000 s^−1^, the high initial resistance of the mayonnaise prevented continuous motor rotation and did not permit stable, repeatable measurements. At the same time, 1000 s^−1^ was the maximum shear-rate condition achievable with the viscometer. Therefore, 1000 s^−1^ represented the only feasible operating condition that provided continuous rotation and repeatable measurements for all four mayonnaise samples. The viscosity measured at this fixed shear rate was used as the reference value for comparison with the corresponding motor current response, ensuring that all samples were evaluated under the same deformation condition.

#### 2.2.3. Current-Based Monitoring Using the Rotating-Bob Viscometer

**Figure 2:**
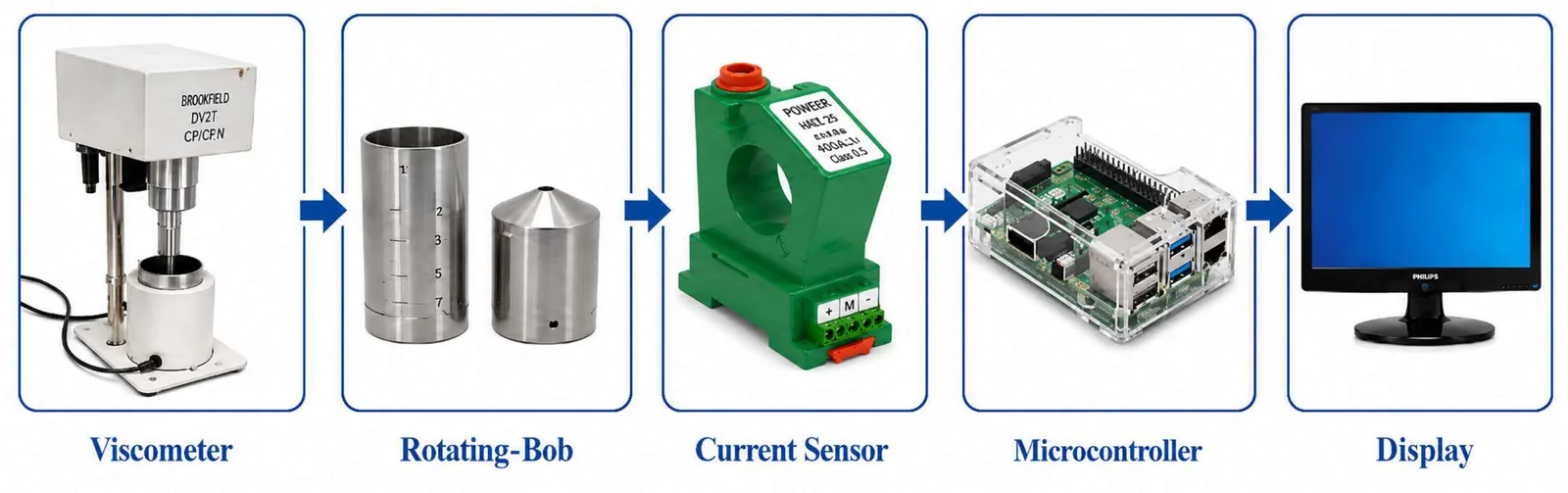
Experimental arrangement for current-based viscosity monitoring using a rotating-bob viscometer, showing the viscometer, rotating-bob assembly, current sensor, microcontroller-based process sensing unit, and display.

In the second stage of the study, the rotating-bob viscometer was integrated with a current sensor and a data-acquisition system. The objective was to determine whether the electrical response of the viscometer motor during fluid shearing could be used to estimate the entropy-related response of the system and relate it to the measured viscosity of the fluids. During rotation, the bob experiences resistance from the surrounding fluid, and this resistance changes the mechanical load applied to the motor. Therefore, the current drawn by the motor was monitored as an indirect measure of the flow resistance offered by each fluid. To record this response, a current sensor was connected to the viscometer motor, and the measured signal was transferred to a microcontroller-based process-sensing unit for real-time acquisition and display, as shown in Figure 2.

#### 2.2.4. Current-Sensor-Based Monitoring During Continuous Shearing of Mayonnaise

**Figure 3:**
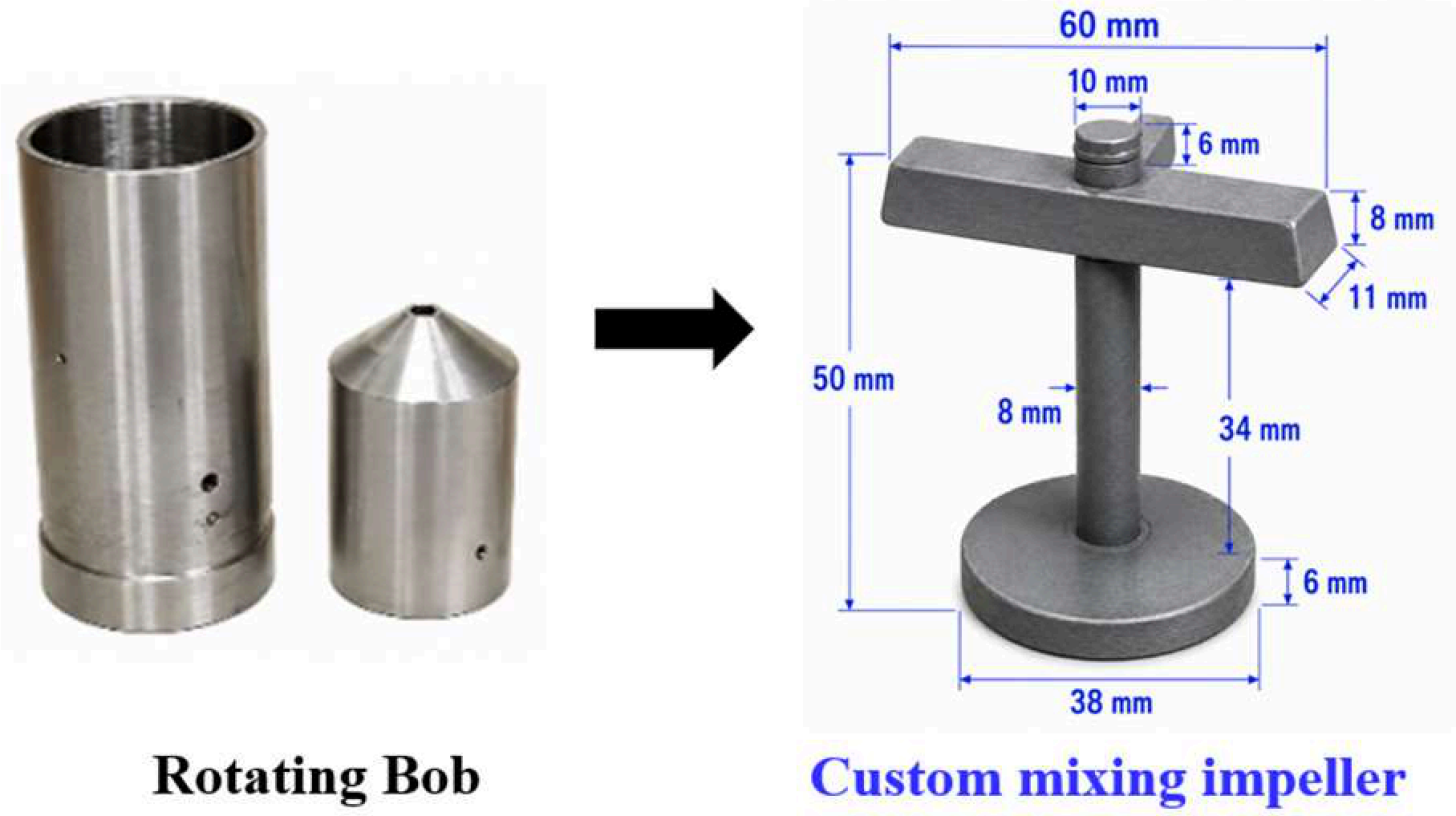
Custom mixing impeller used for continuous laboratory-scale shearing of mayonnaise in the modified viscometer. The principal geometric dimensions are indicated in the figure.

A custom-designed mixing impeller, shown in Figure 3, was attached to the viscometer spindle in place of the rotating-bob assembly. The impeller consisted of a 60 mm-long horizontal paddle, corresponding to a 30 mm radial projection from the rotational axis, an 8 mm-diameter central shaft, a shaft height of approximately 34 mm, and a 38 mm-diameter circular base. The viscometer cup served as the sample container, allowing the impeller to rotate directly within the mayonnaise and impose controlled laboratory-scale mechanical shearing. The geometry was selected to provide repeatable contact and material displacement within the available cup dimensions rather than to reproduce a validated industrial flow field. The impeller-based tests were conducted at the instrument setting corresponding to 1000 s^−1^ in the original bob-and-cup configuration, consistent with the operating condition established in Section 2.2.2.

In this configuration, the setup was not used for direct viscosity measurement, since the standard viscometer calibration is based on the original bob-and-cup geometry. Instead, the modified impeller-based arrangement was used to record the motor current response during mayonnaise shearing. As the impeller rotated through the sample, the resistance of the mayonnaise altered the mechanical load on the motor, and the resulting current was measured using the current sensor. The measured signal was transferred to the microcontroller-based process-sensing unit and displayed in real time. The motor current was recorded continuously from the moment the motor was switched on. During startup, the current increased as the motor accelerated and the impeller engaged with the mayonnaise. This startup ramp was excluded from the analysis, and the peak current reached after impeller engagement was defined as the initial reference current, $I_{0}$. 

To validate the current-based response, mayonnaise samples were collected after selected shearing intervals of 5, 120, 300, 600, 1200, and 1800 s and independently characterized using a rheometer. Accordingly, the first rheometer-measured viscosity point will corresponds to the sample collected after 5 s of shearing. The rheometer used to determine viscosity is described elsewhere [35]. The rheometer-measured viscosity was then compared with the current response recorded during impeller-based shearing. This stage was used to evaluate whether the motor current response could capture the viscosity evolution of mayonnaise under controlled impeller shearing. From the recorded current signal, the corresponding entropy generation response was also calculated.

## 3. Results

### 3.1. Viscosity Measurement of Fluids

**Figure 4:**
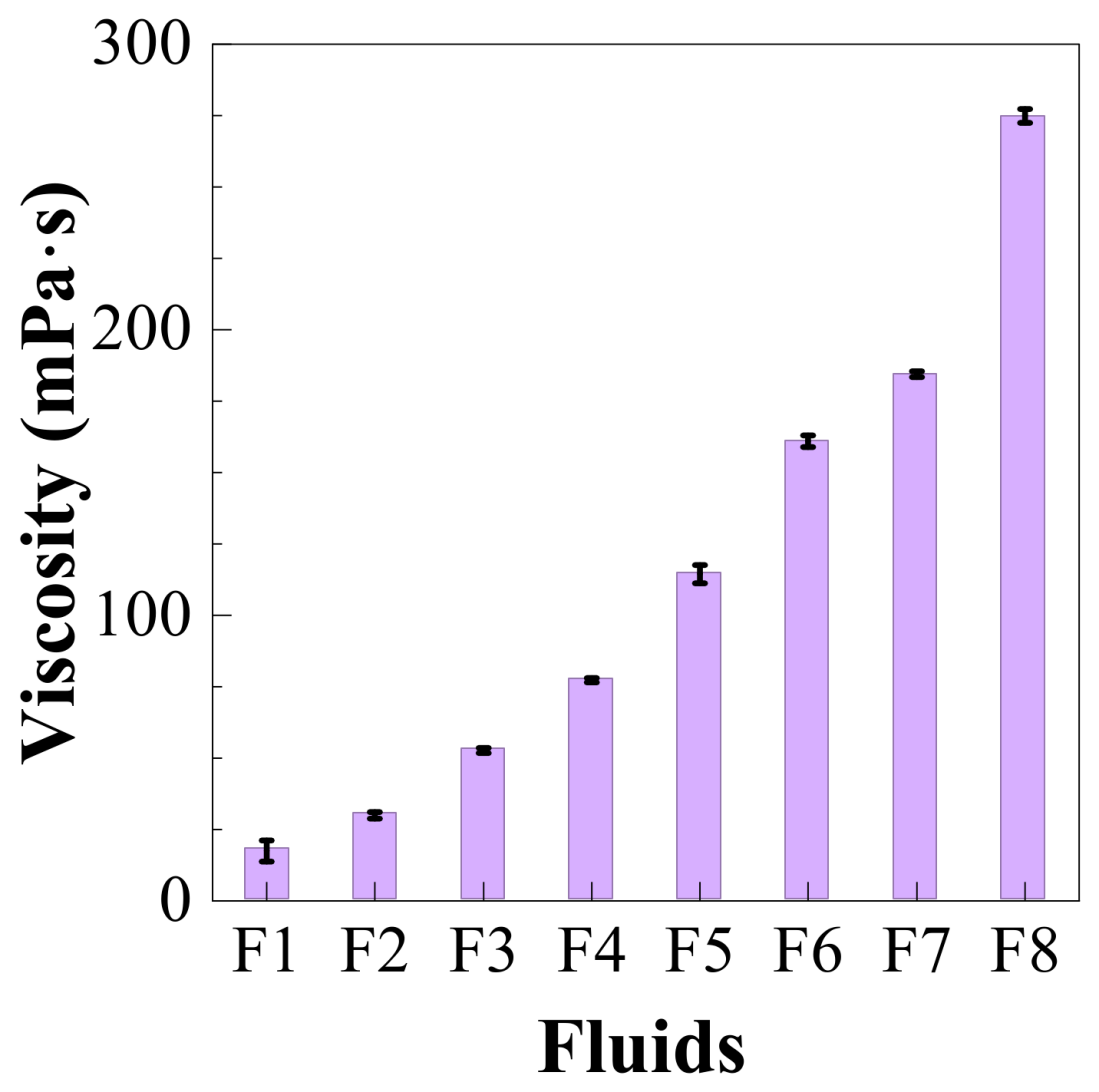
Measured viscosity of eight fluids, F1–F8, showing the viscosity range used for the initial validation study.

The viscosities of eight representative fluids were measured using the rotating-bob viscometer (Figure 1) at shear rates of 600, 800, and 1000 s^−1^. Three independent trials were conducted for each fluid, and the results are reported in Figure 4 as mean ± standard deviation. The error bars represent the experimental variability among the replicate measurements. The measured viscosities showed only minor variation with shear rate for all tested fluids, indicating that the selected samples behaved as Newtonian or near-Newtonian liquids within the tested shear-rate range. Therefore, the viscosity values were not strongly dependent on the operating shear rate, and the average viscosity was used to represent each fluid. The mean viscosities increased progressively from 0.0193 ± 0.0037 Pa·s for F1 to 0.2751 ± 0.0024 Pa·s for F8. F1 exhibited the lowest viscosity and therefore the least resistance to flow, while F8 showed the highest viscosity and the greatest resistance to shearing. Statistical differences among the fluids were evaluated using one-way analysis of variance followed by an appropriate post-hoc comparison, with statistical significance defined at *p* < 0.05.

### 3.2. Correlation Between Motor Current and Fluid Viscosity

**Figure 5:**
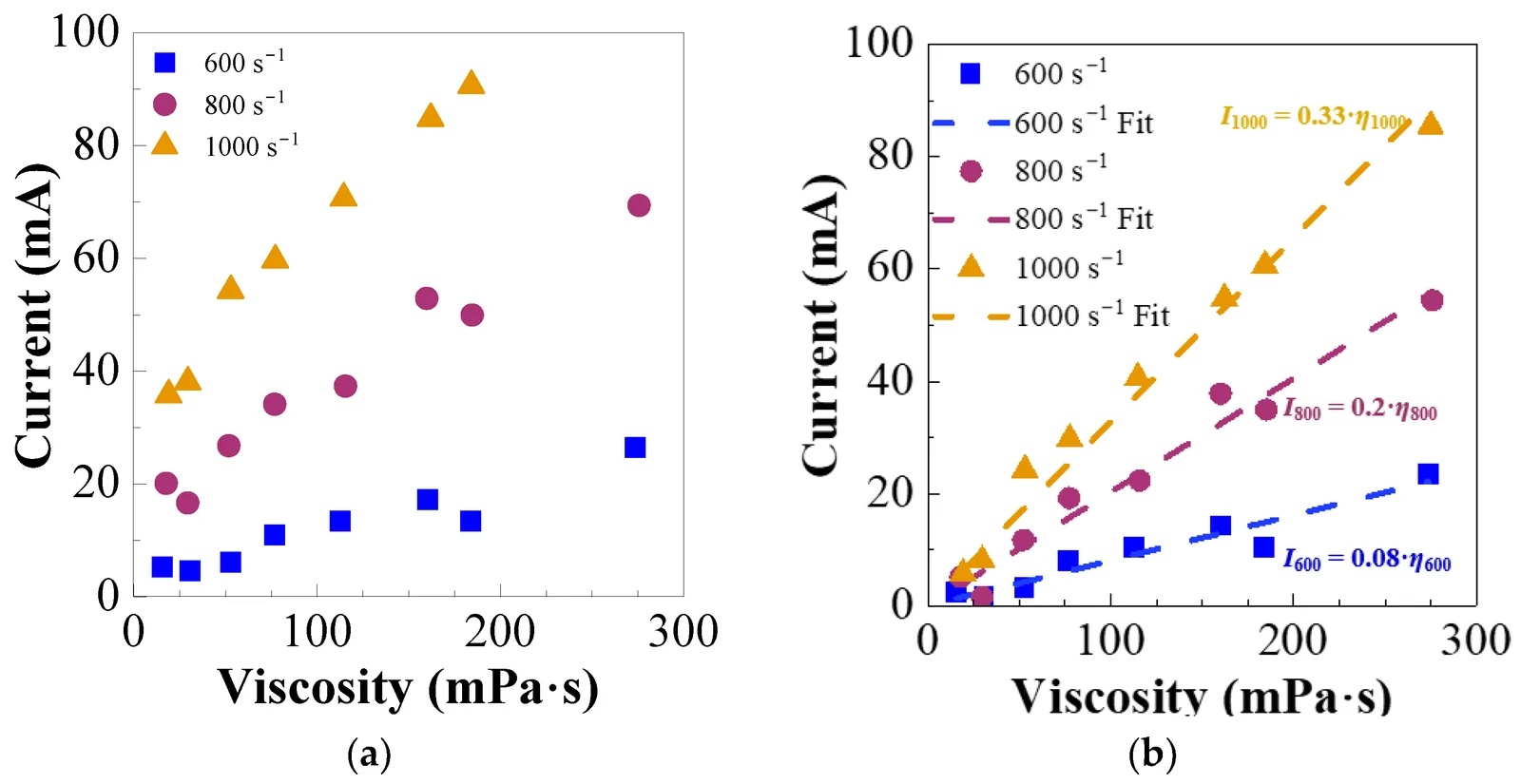
Current-based viscosity response of the fluids using the rotating-bob viscometer. (**a**) Measured motor current as a function of fluid viscosity at shear rates of 600, 800, and 1000 s^−1^. (**b**) Corrected fluid-induced current, obtained by subtracting the corresponding no-load current at each shear rate, plotted against viscosity with linear fitted relationships.

To examine whether the motor current response could serve as an indirect indicator of fluid viscosity, a rotating-bob viscometer was integrated with a current-sensing system (Figure 2). The current drawn by the viscometer motor was measured while each fluid was sheared at three different shear rates: 600, 800, and 1000 s^−1^. Figure 5a shows the measured motor current plotted against the viscosity of the fluids. For all three shear rates, the current generally increased with viscosity, indicating that higher-viscosity fluids offered greater resistance to bob rotation and, consequently, increased the electrical load on the motor. In addition, the current values were higher at higher shear rates, as expected, because increasing the rotational speed increases the mechanical power required to shear the fluid.

However, the measured current in Figure 5a includes both the current required to rotate the viscometer under no-load conditions and the additional current associated with fluid resistance. Therefore, to isolate the contribution of the fluid, the no-load current measured at each shear rate was subtracted from the corresponding total current. The corrected current was calculated as: ${\Delta I}_{\mathrm{fluid}}=I_{\mathrm{measured}}-I_{\mathrm{no}\text{-}\mathrm{load}}$. Here $I_{\mathrm{fluid}}$ is the current contribution associated with shearing the fluid, $I_{\mathrm{measured}}$ is the total current recorded during the test, and $I_{\mathrm{no}\text{-}\mathrm{load}}$ is the baseline current required to rotate the system without fluid loading.

Figure 5b presents the corrected current response as a function of viscosity after subtracting the no-load current. Compared with the raw current data, the corrected current shows a clearer increasing trend with viscosity for all three shear rates. The linear fitted lines show that the current contribution associated with fluid shearing increased approximately linearly with viscosity within the tested range. This indicates that, after subtracting the no-load current, the remaining current response was primarily determined by the fluid’s resistance to the rotating bob. As viscosity increased, a higher mechanical load was imposed on the motor, leading to a corresponding increase in the measured current. The strength of this relationship was further supported by the high regression coefficients obtained at all three shear rates. The $R^{2}$ values for the fitted relationships at 600, 800, and 1000 s^−1^ were 0.97, 0.99, and 0.995, respectively. The improvement in $R^{2}$ at higher shear rates suggests that the current response became more strongly correlated with viscosity as the shearing intensity increased. The linear fit equations determined for 600, 800, and 1000 s^−1^ are *I*_600_ = 0.08·*η*_600_, *I*_800_ = 0.203·*η*_800_, and *I*_1000_ = 0.328·*η*_1000_. These results show that the corrected motor current response is highly sensitive to fluid viscosity and can be considered as an indirect indicator of rheological resistance during rotating-bob shearing.

### 3.3. Current and Viscosity Monitoring for Mayonnaise Using a Viscometer

**Figure 6:**
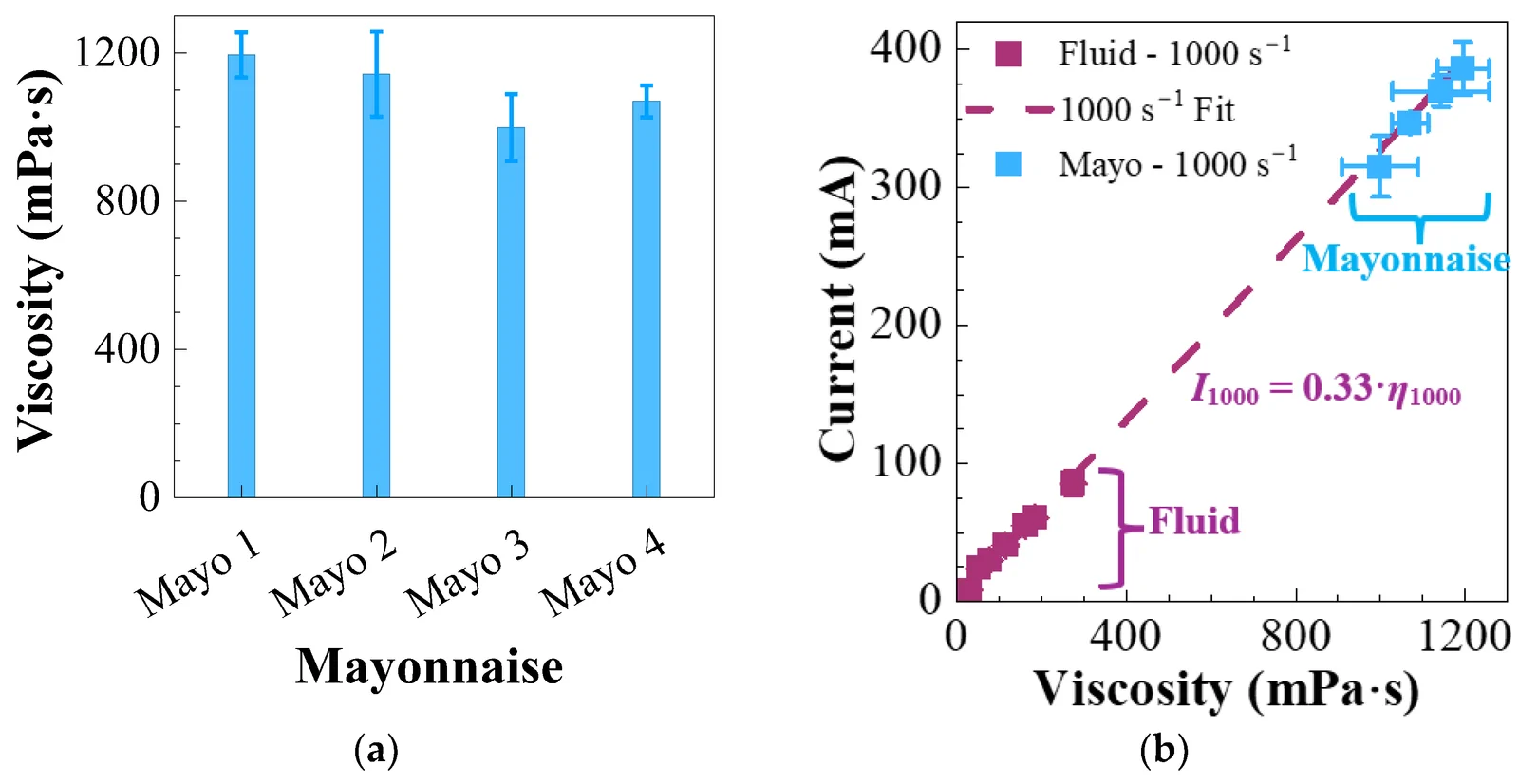
Extension of current-based viscosity monitoring to mayonnaise samples. (**a**) Rheometer-measured viscosity of four mayonnaise samples at a shear rate of 1000 s^−1^. (**b**) Comparison of mayonnaise current response with the current–viscosity relationship obtained from the initial fluids at 1000 s^−1^, showing the alignment of the mayonnaise samples with the extrapolated fluid-based trend.

After establishing the relationship between viscosity and motor current response for the initial oil-based fluids, the same approach was extended to four commercially available mayonnaise samples. Their viscosities were measured at a shear rate of 1000 s^−1^ in three independent trials, and the results are presented in Figure 6a as mean ± standard deviation. Since mayonnaise is a shear-sensitive emulsion and its viscosity decreases with continued shearing, the initial viscosity reading at 1000 s^−1^ was used for the analysis. This value represents the early-stage shear resistance of each sample and was compared with the corresponding motor current response measured under the same operating condition. The measured viscosities were 1.195 ± 0.060, 1.143 ± 0.114, 0.999 ± 0.090, and 1.069 ± 0.043 Pa·s for Mayo 1, Mayo 2, Mayo 3, and Mayo 4, respectively. One-way analysis of variance showed that the differences among the four mayonnaise samples were not statistically significant at *p* < 0.05 (F = 3.32, *p* = 0.078). This confirmed that the mayonnaise samples provided a higher-viscosity food emulsion, enabling evaluation of whether the current-based approach could be extended beyond lower-viscosity oil samples.

Figure 6b compares the current response of the mayonnaise samples with the relationship previously obtained for the initial fluids at 1000 s^−1^, i.e.,$\;I_{1000}=0.328\cdot \eta_{1000}$. The data points are presented with horizontal and vertical error bars representing the standard deviations of viscosity and motor current, respectively, from three independent trials. The fitted relationship derived from the initial fluid data was extended into the higher-viscosity range and compared with the mean current values measured for the mayonnaise samples. The mayonnaise data points aligned well with the extrapolated trend, indicating that the motor current response continued to increase with viscosity, even in the higher-viscosity emulsion samples. Linear regression across the complete viscosity range showed a strong and statistically significant relationship between viscosity and motor current ($R^{2}=0.99$, p<0.001). This suggests that the current drawn by the motor remains sensitive to the resistance offered by the material during shearing.

### 3.4. Accumulated Entropy Generation (AEG)

**Figure 7:**
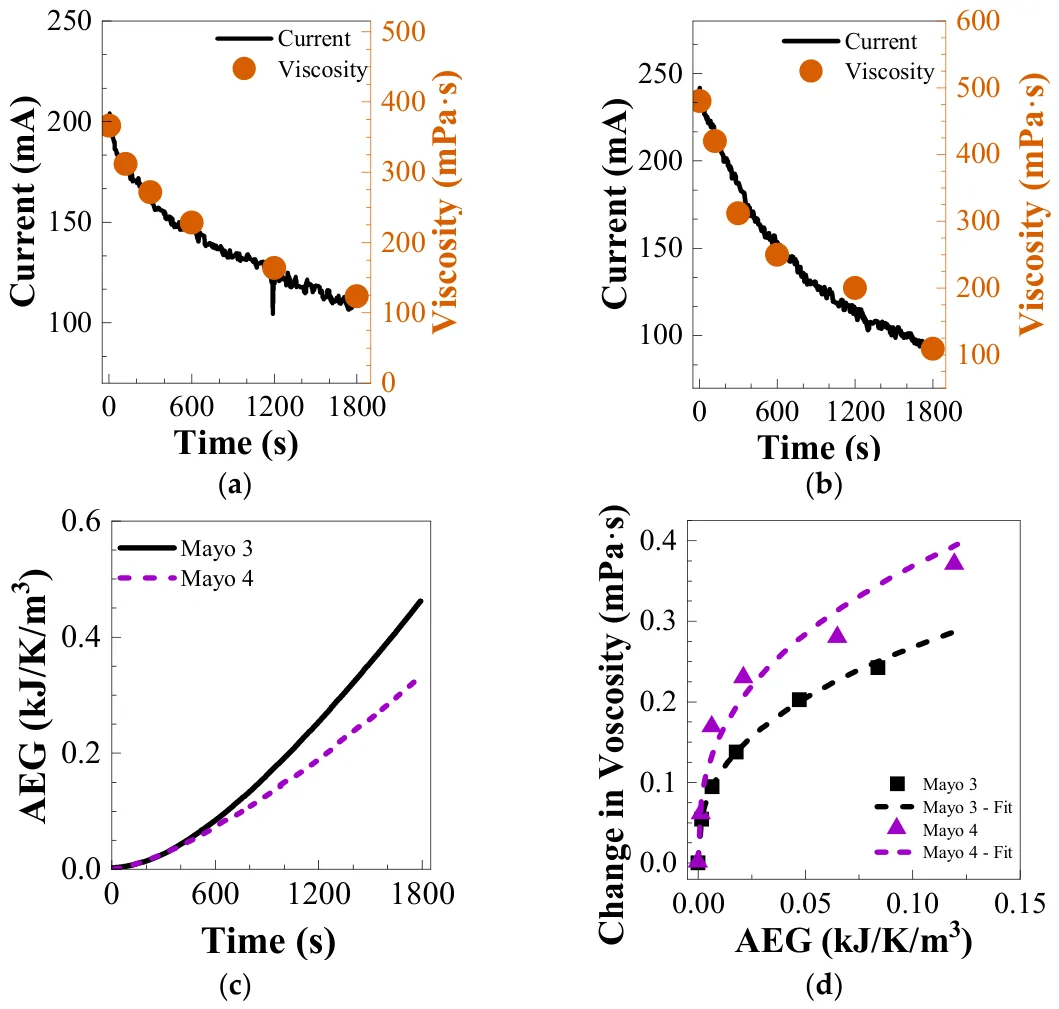
Time-dependent current, viscosity, and accumulated entropy-generation response of Mayo 3 and Mayo 4 during impeller-based shearing. (**a**,**b**) Continuous motor current response measured using the modified viscometer and corresponding rheometer-measured viscosity values obtained at selected shearing times for Mayo 3 and Mayo 4 at 1000 s^−1^. (**c**) Accumulated entropy generation calculated from the current response. The higher AEG response of Mayo 4 indicates greater structural evolution during shearing, consistent with its larger reduction in viscosity and observed oil separation. (**d**) Relationship between accumulated entropy generation and viscosity change, showing the shear-induced degradation response of Mayo 3 and Mayo 4. Mayo 4 reached the same viscosity change at a lower AEG than Mayo 3, indicating faster structural degradation under the same shearing condition.

All four mayonnaise samples were included in the initial current–viscosity evaluation presented in Section 3.3. Mayo 3 and Mayo 4 were subsequently selected for the time-dependent AEG and DEG analyses because they were obtained from the same manufacturer while differing in oil-phase composition. This selection enabled a more controlled comparison of formulation-dependent shear behavior by minimizing variability associated with differences between manufacturers. Mayo 3 was palm-oil-based, whereas Mayo 4 was olive-oil-based. The two samples were sheared using the modified impeller-based viscometer setup under the operating condition corresponding to 1000 s^−1^, while the motor current was continuously monitored with time. At selected shearing intervals, samples were collected and independently tested using a rheometer to determine the corresponding viscosity at the same shear-rate condition. This allowed the continuous motor current response from the viscometer to be compared with discrete rheometer-based viscosity measurements during mayonnaise shearing. Figure 7a,b show the time-dependent current and viscosity responses of Mayo 3 and Mayo 4, respectively. For both samples, the motor current decreased with shearing time, indicating a reduction in the resistance experienced by the rotating impeller. This trend was consistent with the rheometer measurements, which also showed a decrease in viscosity during shearing. However, the two samples exhibited different levels of structural response. Mayo 3 showed a lower initial current and lower initial shearing resistance, and its viscosity decreased only moderately with time. This suggests that the palm-oil-based mayonnaise experienced a comparatively smaller structural change under the applied shearing conditions.

In contrast, Mayo 4 exhibited a higher initial current and viscosity, indicating greater resistance to impeller rotation at the start of the test. With continued shearing, Mayo 4 showed a more pronounced reduction in both current and viscosity, suggesting greater structural breakdown. Visible oil separation was also observed for Mayo 4 during/after shearing, indicating partial destabilization of the emulsion structure under mechanical deformation. Therefore, the current response was able to capture not only the reduction in flow resistance but also the difference in shear stability between the two mayonnaise formulations. The AEG was estimated using Equation (5), and the obtained results are shown in Figure 7c. For both mayonnaise samples, AEG increased continuously with shearing time, indicating the progressive accumulation of irreversible mechanical dissipation during processing. However, the magnitude of AEG differed between the two samples. Mayo 4 generated a higher accumulated entropy response, consistent with its larger reduction in current and viscosity and the observed oil separation. This indicates greater cumulative dissipative change and greater apparent structural evolution during shearing. In comparison, Mayo 3 showed a lower AEG response, reflecting its smaller viscosity change and comparatively more stable behavior under the same shearing condition.

To examine the relationship between accumulated dissipation and rheological change, the viscosity-change parameter defined in Section 2.1 was plotted against AEG, as shown in Figure 7d. Both Mayo 3 and Mayo 4 exhibited a transition-type degradation response. In the initial stage, a small increase in AEG produced a relatively rapid change in viscosity, indicating that the mayonnaise structure was highly sensitive to early-stage shearing. After this transient region, the rate of viscosity change became more gradual, suggesting that the system approached a more stable shearing response.

A clear difference was observed between the two mayonnaise samples. Mayo 4 showed a larger viscosity change than Mayo 3 for the same level of AEG, indicating that the olive-oil-based mayonnaise was more sensitive to mechanical shearing and experienced faster structural degradation. In contrast, Mayo 3 showed a lower Δη at the same AEG level, suggesting greater resistance to shear-induced viscosity loss. For example, to achieve a viscosity change of 0.25 Pa·s, Mayo 3 required an AEG of approximately 0.27 kJ/K/m^3^, whereas Mayo 4 required only about 0.19 kJ/K/m^3^. This indicates that Mayo 3 required greater accumulated entropy generation to reach the same level of rheological transformation, while Mayo 4 reached the same change at a lower AEG. Therefore, the Δη–AEG response confirms that Mayo 4 degraded more rapidly under the same shearing condition.

### 3.5. Degradation Coefficient (B)

**Figure 8:**
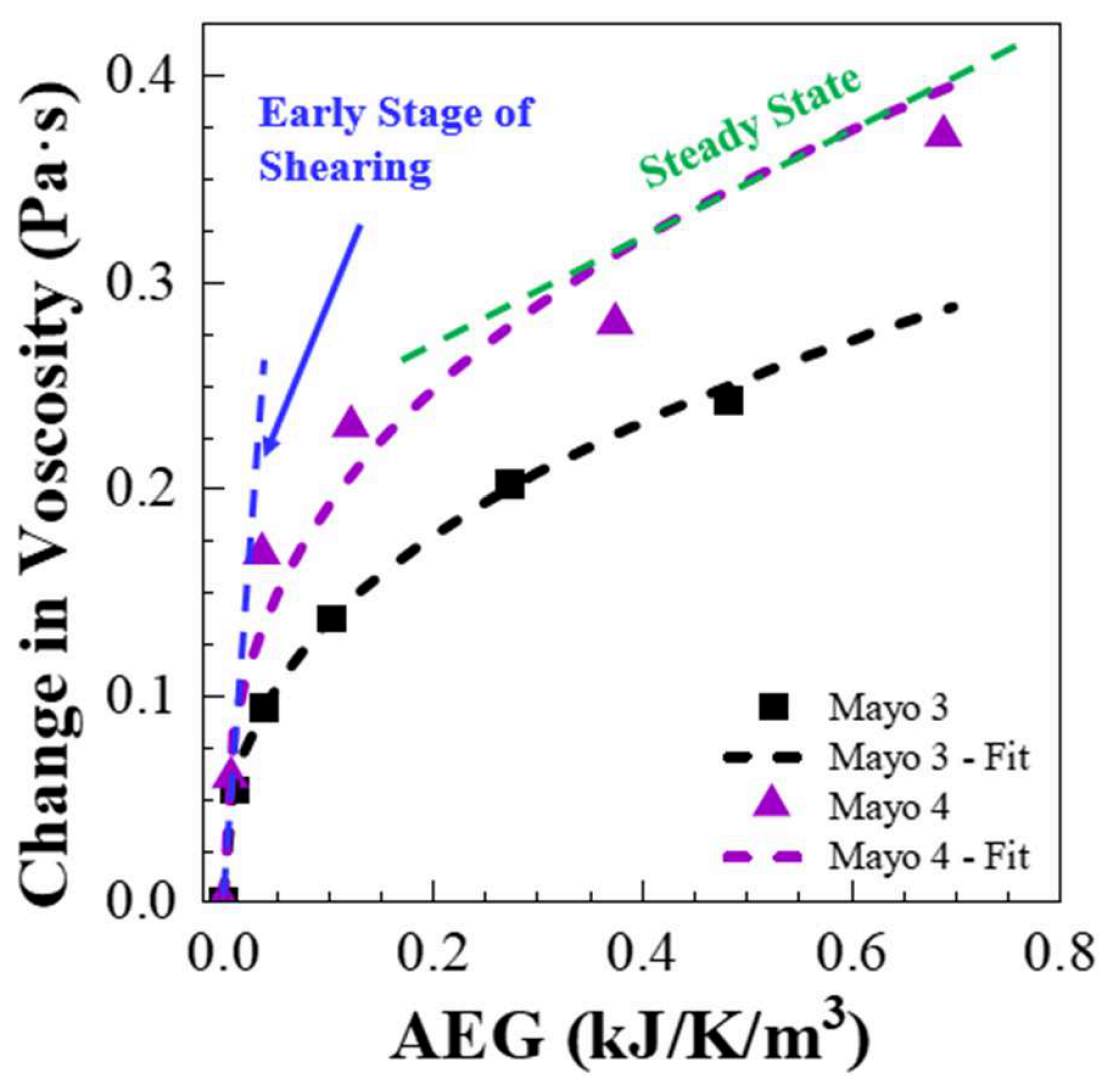
Relationship between accumulated entropy generation (AEG) and viscosity-based degradation of Mayo 3 and Mayo 4 during shearing. The change in viscosity, $\Delta \eta /\eta_{0}$, was used as a damage indicator. The fitted curves show an initial rapid degradation region followed by a slower steady-state degradation regime. Mayo 4 exhibited a higher degradation response than Mayo 3, indicating greater viscosity loss per unit AEG and lower shear stability under the same shearing condition.

The effective viscosity-based degradation coefficient, B, was determined from the slope of the normalized viscosity-change–AEG relationship defined in Equation (9). Because the Δη–AEG response was nonlinear (Figure 8), the *B* coefficient was evaluated separately in two regions: the early stage of shearing and the steady-state degradation region. The initial region represents the rapid structural breakdown that occurs at the onset of shearing, while the steady-state region represents the slower degradation stage after the material has undergone significant structural rearrangement.

A distinct difference in the B coefficient was observed between Mayo 3 and Mayo 4. Mayo 4 exhibited higher B values than Mayo 3 in both the initial and steady-state regions, indicating a greater viscosity loss per unit accumulated entropy generation. In the initial shearing stage, the B coefficient for Mayo 3 was approximately 2.5 × 10^−2^ K·m^3^/kJ, whereas Mayo 4 showed a higher value of 3.0 × 10^−2^ K·m^3^/kJ. In the steady-state region, the corresponding values were $7.9\times 10^{-4}$ and $8.1\times 10^{-4}\;K\;m^{3}/kJ$ for Mayo 3 and Mayo 4, respectively. From the DEG perspective, these results indicate that Mayo 4 degraded at a faster rate and had lower shear stability than Mayo 3. The initial-stage B coefficients were also substantially higher than the steady-state values for both samples. This confirms that the most rapid viscosity degradation occurred during the early stage of shearing, when the emulsion structure was more sensitive to mechanical deformation. After this transient region, the lower steady-state B values suggest that the samples entered a slower and more gradual degradation regime under continued shearing.

These results support the earlier observation that Mayo 4 underwent faster structural breakdown during shearing. The higher degradation coefficient of Mayo 4 is consistent with its larger viscosity reduction, higher AEG response, and observed oil separation. In contrast, the lower B values for Mayo 3 indicate greater resistance to shear-induced viscosity loss. Therefore, the DEG-based B coefficient provides an effective quantitative measure for comparing the shear stability of different mayonnaise formulations.

## 4. Discussion

The results show that motor current can track changes in mayonnaise resistance during shearing. For both Mayo 3 and Mayo 4, the measured current and rheometer-measured viscosity decreased with time, indicating progressive structural weakening under continued mechanical deformation. This agreement supports the use of motor current as an indirect indicator of viscosity evolution during mayonnaise shearing. The AEG response provides an interpretation beyond current or viscosity alone. Current represents the instantaneous electrical response of the motor, while viscosity represents the rheological state measured at selected time points. In contrast, AEG accumulates the dissipative history of the process. Therefore, AEG provides a way to describe not only the current state of the sample but also the mechanical history experienced during shearing. This is important for mayonnaise because its structure depends strongly on shear history, droplet rearrangement, and possible emulsion destabilization.

The relationship between AEG and viscosity change further shows that the mayonnaise samples followed a transition-type degradation response. An initial transient region was followed by a steady-state degradation region, suggesting that the structure was more sensitive to early-stage shearing and then approached a more stable degradation rate. This type of transient-to-steady-state degradation behavior is similar to that of mechanically worked grease systems, where shearing initially causes rapid structural breakdown, followed by a slower degradation regime under continued working [35,37]. Similar entropy-based relationships have also been observed for the transient wear of tribo-pairs [38,39,40].

The effective viscosity-based degradation coefficient, B, complements the AEG trajectories by expressing the apparent viscosity loss produced per unit current-derived AEG. Its variation between the initial and quasi-steady regions indicates that the response depends on the evolving structural state of the mayonnaise rather than remaining constant throughout continuous shearing. The higher values obtained for Mayo 4 further identify its greater susceptibility to shear-induced structural change relative to Mayo 3.

A limitation of the present study is that the experiments were conducted using a laboratory-scale, modified viscometer without active temperature control or independent characterization of the flow field generated by the custom mixing impeller. A formal experimental sensitivity analysis of temperature and sample volume was therefore not conducted. Consequently, the current-derived AEG values should be interpreted as comparative descriptors obtained under fixed experimental conditions rather than as absolute industrial process measurements. Future studies should incorporate controlled temperature and volume variation, direct power calibration, and computational or experimental flow-field characterization before establishing scale-up relationships with industrial mixing equipment.

The degradation coefficient, *B*, was evaluated only at the fixed operating condition used in the present setup; consequently, its dependence on shearing intensity remains to be established using equipment capable of stable operation across multiple controlled conditions. Pilot- and plant-scale validation should simultaneously monitor motor current, voltage, temperature, mixing speed, batch volume, ingredient-addition sequence, and final product attributes. Relating the resulting AEG trajectories to viscosity, emulsion stability, oil separation, texture, and product acceptability could support the development of reference trajectories for batch monitoring and early identification of process deviations.

## 5. Conclusions

This study demonstrates the feasibility of using motor current-derived accumulated entropy generation (AEG) to monitor shear-induced changes in mayonnaise. Reference-fluid tests showed a strong linear relationship between corrected motor current and viscosity, confirming that the current reflects the material’s resistance during shearing. For mayonnaise, the motor current decreased with shearing time in agreement with the rheometer-measured viscosity reduction. The calculated AEG captured the cumulative dissipative history of the samples and distinguished their shear stability. Mayo 4 reached the same viscosity degradation at a lower AEG than Mayo 3, indicating faster structural breakdown and lower shear stability. The DEG-based B coefficient further confirmed this trend, with Mayo 4 showing higher degradation coefficients than Mayo 3 in both the initial and steady-state shearing regions. These results support current-derived entropy parameters (*B* coefficient and AEG) as a promising comparative thermodynamic descriptor for monitoring mayonnaise consistency evolution. Future work should validate the approach in pilot- or plant-scale mayonnaise production and relate AEG trajectories to final product quality.

## 6. Patents

Fluid Monitoring (for Food), US Patent, US 12,298,293 B2, M. M. Khonsari, K.P. Lijesh, Granted-2025.

## Figures and Tables

**Table 1:** Measured viscosity of selected fluids using the rotating bob viscometer.

Fluid Code	Representative Description	Original Fluid Type
F1	Low-viscosity paraffinic hydrocarbon oil	Mineral oil
F2	Glycol-based heat-transfer fluid	Coolant
F3	Triglyceride-based vegetable oil	Sunflower oil
F4	Vegetable-oil-modified lubricant blend	SAE40 + sunflower oil
F5	Newtonian viscosity reference liquid	100 cP standard
F6	Medium-viscosity monograde lubricant	SAE40 oil
F7	Mixed-grade lubricant blend	SAE50 + SAE40
F8	High-viscosity monograde lubricant	SAE50 oil

## Data Availability

Data will be provided on request.

## Nomenclature

The following nomenclature are used in this manuscript:

AEG	Accumulated entropy generation
rpm	Revolutions per minute
I(t)	Time-dependent motor current
$I_{0}$	Initial motor current
ΔI(t)	Change in motor current
V	Motor voltage
Vol	Sample volume
P(t)	Time-dependent power input
T	Absolute temperature
η	Viscosity
$\eta_{0}$	Initial viscosity
η(t)	Time-dependent viscosity
Δη	Change in viscosity
τ	Shear stress
$\dot{\gamma }$	Shear rate
